# STEAP4 expression in CNS resident cells promotes Th17 cell-induced autoimmune encephalomyelitis

**DOI:** 10.1186/s12974-021-02146-7

**Published:** 2021-04-20

**Authors:** Junjie Zhao, Yun Liao, William Miller-Little, Jianxing Xiao, Caini Liu, Xiaoxia Li, Xiao Li, Zizhen Kang

**Affiliations:** 1 Department of Inflammation and Immunity, Lerner Research Institute, Cleveland, OH USA; 2 The Center for RNA Science and Therapeutics, Case Western Reserve University, Cleveland, OH USA; 3 Department of Pathology, University of Iowa, Iowa City, IA 52242 USA

**Keywords:** Steap4, Th17, Interleukin-17, EAE, Multiple sclerosis

## Abstract

**Background:**

Multiple sclerosis (MS) is a debilitating neurological disease caused by autoimmune destruction of the myelin sheath. Experimental autoimmune encephalomyelitis (EAE) is a widely used animal model for the pathogenesis of MS. We and others have previously demonstrated that IL-17 is critical for the pathogenesis of EAE. The concentration of IL-17 is significantly higher in the sera of MS patients than in healthy controls and correlates with disease activity. Moreover, anti-IL-17 neutralizing antibody demonstrated promising efficacy in a phase II trial in MS patients, further substantiating a key pathogenic role for IL-17 in MS. While Th17 and IL-17 are emerging as a bona fide drivers for neuroinflammation, it remains unclear what effector molecule executes the inflammatory tissue destruction in Th17-driven EAE.

**Methods:**

By microarray analysis, we found STEAP4 is a downstream molecule of IL-17 signaling in EAE. We then used STEAP4 global knockout mice and STEAP4 conditional knockout mice to test its role in the pathogenesis of EAE.

**Results:**

Here, we report that the metalloreductase, STEAP4, is a key effector molecule that participates and contributes to the pathogenesis of Th17-mediated neuroinflammation in experimental autoimmune encephalomyelitis. *STEAP4* knockout mice displayed delayed onset and reduced severity of EAE induced by active immunization. The reduced disease phenotype was not due to any impact of STEAP4 deficiency on myelin reactive T cells. In contrast, *STEAP4* knockout mice were resistant to passively induced EAE, pointing to a role for STEAP4 in the effector stage of EAE. Notably, *STEAP4* was only induced the spinal cord of EAE mice that received Th17 cells but not Th1 cells. Consistently, STEAP4 deficiency protected from only Th17 but not Th1-induced EAE. Finally, using Nestin-Cre STEAP4^fl/fl^ mice, we showed that ablation of STEAP4 expression in the resident cells in the central nervous system attenuated disease severity in both active immunization and passive Th17 transfer-induced EAE.

**Conclusion:**

In this study, we identified STEAP4 as a Th17-specific effector molecule that participates and contributes to the pathogenesis of neuroinflammation, thus potentially provide a novel target for MS therapy.

**Supplementary Information:**

The online version contains supplementary material available at 10.1186/s12974-021-02146-7.

## Introduction

Experimental autoimmune encephalomyelitis (EAE) is a widely used animal model for the pathogenesis of multiple sclerosis (MS), a debilitating neurological disease caused by autoimmune destruction of the myelin sheath [1–3]. While T helper cells were shown to drive the neuroinflammation in the EAE model in 1980s, significant progresses in elucidating the pathogenic mechanism were made predominantly in the past two decades [4–6]. The discovery of Th17, a unique T helper cell subset that produces IL-17, led to a major realignment of our understanding of the immunopathogenic process [7–9]. While myelin-reactive Th1 and Th17 cells can both mediate encephalomyelitis when they are transferred into sublethally irradiated mice, abrogation of Th17 cell differentiation renders mice resistant to the induction of EAE by active immunization [9, 10], highlighting a more fundamental role for Th17 lineage cells in a normal course of autoimmune pathogenesis.

As the signature cytokine of Th17 cells, IL-17 subsequently became the focus of study as the culprit cytokine that drives neuroinflammation in EAE [11–13]. Prevailing evidence indicate that IL-17 is required for the pathogenesis of EAE in mice models. Mice deficient in IL-17 or IL-17 receptor are protected from active immunization-induced EAE [11–13]. IL-17 binds a heterodimer receptor complex, which then transduces the signal through an adaptor molecule, Act1 [14, 15]. Taking advantage of the critical role for Act1 in IL-17 signaling, we have previously completed and reported a series of studies examining the cell-type specific role of IL-17 signaling in EAE pathogenesis. These efforts have identified the resident cells in the central nervous systems (CNS), especially the NG2+ oligodendrocyte progenitor cells, as the primary target of IL-17 and critical drivers for Th17-mediated EAE [16, 17]. In agreement with the findings from the EAE model, correlative studies using patient specimen have strongly implicated IL-17 in the pathogenesis of MS. The concentration of IL-17 is significantly higher in the sera of MS patients than in healthy controls and correlates with disease activity. Moreover, anti-IL-17 neutralizing antibody demonstrated promising efficacy in a phase II trial in MS patients, further substantiating a key pathogenic role for IL-17 in MS [18].

While Th17 and IL-17 are emerging as a bona fide drivers for neuroinflammation, it remains unclear what effector molecule executes the inflammatory tissue destruction in Th17-driven EAE. Addressing this question would be crucial for our understanding of the underlying process, as myelin-reactive Th1 cells are equally potent at mediating demyelination, yet Th1 lineage cells are dispensable during active EAE [9]. To identify effector molecules that mediate Th17-driven neuroinflammation, we performed transcriptomic profiling of spinal cord tissue from EAE mice that received myelin-reactive Th17 or Th1 cells. Differential gene expression analysis identified *STEAP4*, as a target gene that was highly induced in Th17- but not Th1-induced EAE mice.

*STEAP4* (six-transmembrane epithelial antigen of the prostate) encodes a transmembrane protein that consist of an N-terminal cytoplasmic oxidoreductase domain (OxRD) and a six-helical transmembrane domain (TMD). STEAP family proteins were originally cloned from prostate cancer cells [19] and were later found to exhibit metalloreductase activity that catalyzes iron(III) and copper (II) reduction using intracellular NADPH [20–23]. Elevated expression of STEAP4 has been linked to pathogenesis of intestinal inflammation [24], cancer [25, 26], and metabolic diseases [27]. In this study, we sought to investigate the role of *STEAP4* as an effector molecule in the pathogenesis of Th17-driven EAE.

## Materials and methods

### Mice

All mice used in the experiment were housed under specific pathogen-free conditions. STEAP4-deficient mice and STEAP4^floxed/floxed^ mice in the C57BL/6J background were generated as described [25, 28]. B6.Cg-Tg(Nes-Cre)1Kln/J (NesCre transgenic )[29, 30], C57BL/6J were purchased from Jackson laboratory. For all experiments, mice were 6–12 weeks old, age-matched littermates between experimental groups. Experimental protocols were approved by the Institutional Animal Care and Use Committee of Cleveland Clinic.

### Transcriptomic profiling of EAE spinal cord

The transcriptomic profiling experiment that led to the identification of STEAP4 as a target gene in Th17-induced EAE was first described in our previous study [31]. Briefly, five mice in each group were left untreated (naive group) or transferred with MOG-reactive Th1 or Th17 cells. At peak of disease, the mice were killed, and spinal cords were harvested for mRNA extraction, followed by microarray analysis. The samples was hybridized on an Affymetrix GeneChip HT-MG-430PM-96 (Affymetrix). Three independent biological replicates were analyzed in each experiment, which yielded consistent results. The dataset is deposited the Gene Expression Omnibus under accession code GSE97035.

### Induction and assessment of EAE

Active EAE and passive EAE was induced and assessed as previously described [14, 17]. In brief, for adoptive transfer (passive EAE induction), donor mice were subcutaneously immunized with MOG_35–55_ peptide. Then draining lymph nodes and spleen were harvested 10 days after immunization. To prepare MOG_35–55_-specific polarized T cells, donor mice were immunized with MOG_35-55_ subcutaneously, draining lymph node cells were prepared from donor mice 10 days after immunization. Cells were cultured for 5 days with MOG_35–55_ at a concentration of 10 μg/ml under Th1 (20 ng/ml rmIL-12[R&D], 2 μg/ml αIL-23p19 [eBioscience]) polarizing conditions or Th17 (20 ng/ml rmIL-23[R&D]) polarizing condition. Each recipient mouse was injected with 3.0 × 10^7^ MOG_35–55_-specific Th1 or Th17 cells 4 h after 500-Rad sub-lethal irradiation. Clinical scores were assessed in double-blind manner.

### Isolation of CNS inflammatory cells and flow cytometry

Brains were homogenized in ice-cold tissue grinders, filtered through a 100-μm cell strainer and the cells collected by centrifugation at 400×*g* for 5 min at 4 ^°^C. Cells were resuspended in 10 ml of 30% Percoll (Amersham Bioscience) and centrifuge onto a 70% Percoll cushion in 15-ml tubes at 800×*g* for 30 min. Cells at the 30–70% interface were collected and were subjected to flow cytometry. Fluorescence-conjugated CD4 (Clone GK1.5), CD8 (Clone 53-6.7), CD45 (Clone 30-F11), Ly6G (Clone 1A8) monoclonal antibodies and isotype controls were purchased from BD Biosciences. F4/80(Clone Cl: A3-1) was obtained from Serotech. The antibodies were diluted at 1:100 when used. Flow cytometry data were analyzed by FlowJo software.

### Histological analysis

For paraffin-embedded tissue spinal cord removed from PBS perfused mice were fixed in 10% formalin. Sections were stained with either hematoxylin and eosin (H&E) or myelin basic protein (MBP) to evaluate inflammation and demyelination, respectively. For frozen sections, spinal cords were embedded in OCT (Tissue-Tek) and snap frozen on liquid nitrogen. Sections (10 μm of thickness) were incubated with anti-CD4 antibody (BD Biosciences). Antigens were visualized following incubation with fluorescence-conjugated secondary antibodies (Molecular probe).

### ELISA

IL-17 and IFN-γ level were assayed by IL-17- or IFN-γ ELISA kit (R&D systems) following the manufacture’s instruction.

### Real-time PCR

Total RNA was extracted from spinal cord and cultured astrocytes with TRIzol (Invitrogen) according to the manufacture’s instruction. All gene expression results are expressed as arbitrary units relative to expression of the gene encoding β-actin. Fold induction of gene expression in spinal cord after EAE induction was determined by dividing the relative abundance of experimental samples by the mean relative abundance of control samples from naïve mice.

### Statistics

Non-parametric statistics was applied to all data set. The *p* values of clinical scores were determined by two-way multiple-range analysis of variance (ANOVA) for multiple comparisons. Other *p* values were determined by Mann-Whitney test unless otherwise specified. A *p* value of < 0.05 was considered significant. Unless otherwise specified, all results are shown as mean and the standard error of the mean (mean ± SEM).

## Results

### *STEAP4* promotes the pathogenesis of autoimmune encephalomyelitis

**Fig. 1:**
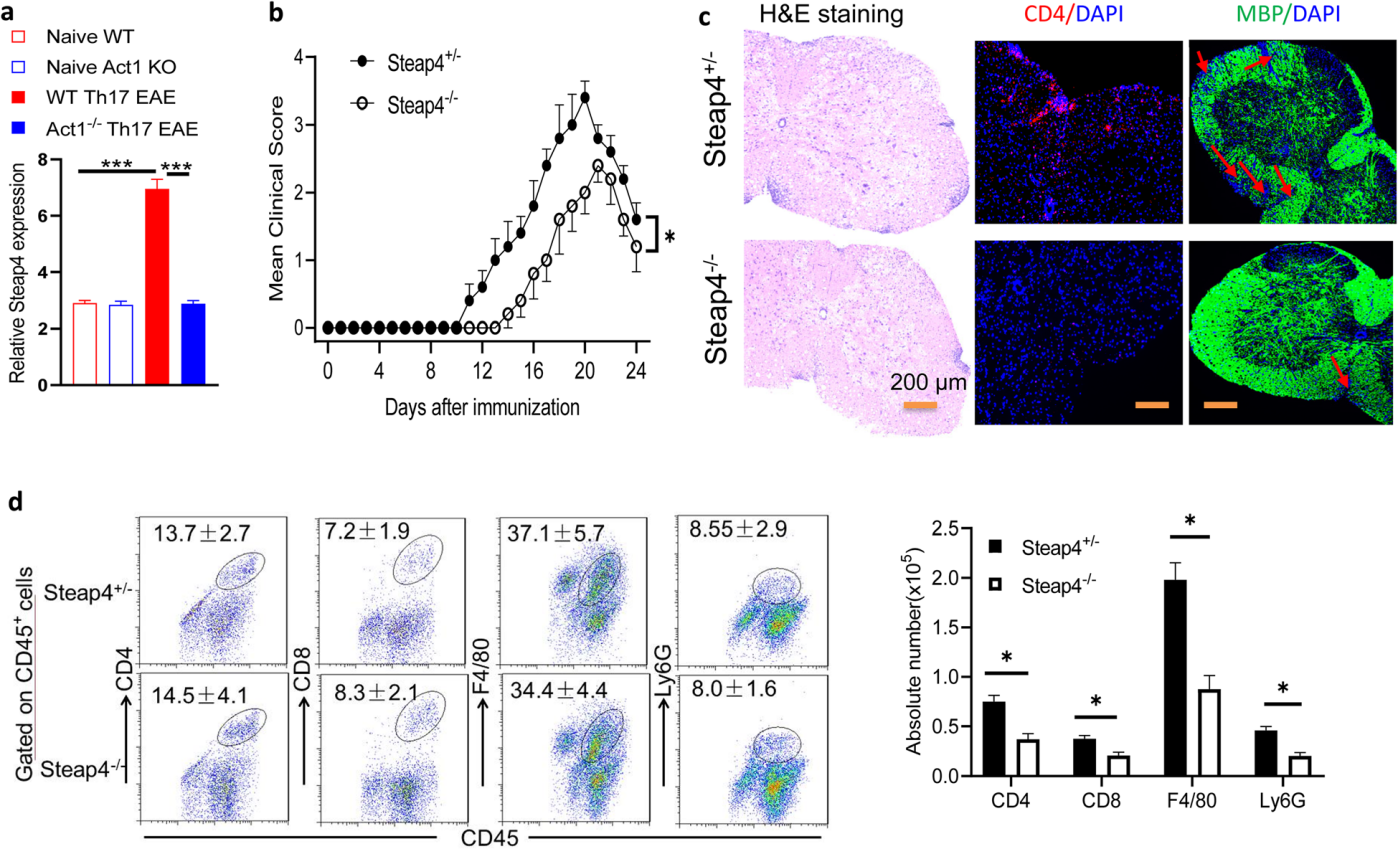
Steap4 promotes actively-induced EAE. **a** Microarray analysis of STEAP4 expression in spinal cords from indicated mice that received MOG_35–55_-reactive Th17 cells and at the peak of Th17-driven EAE, relative to the naïve control mice. Data are re-plotted from GSE97035. **b** Mean clinical score of EAE in STEAP4^+/−^ and Steap4^+/−^ mice induced by active immunization with MOG_35–55_ (*p* < 0.05, two-way ANOVA). **c** H&E, anti-CD4, and myelin basic protein (MBP) staining of lumber spinal cords 20 days after immunization (scale bar, 200 μm). **d** Absolute cell numbers of infiltrated immune cells in the brains of STEAP4^+/−^ and STEAP4^+/−^ mice 20 days after immunization. Data are representative of three independent experiments. *n* = 5/group in each experiment. Error bars, SEM; *p < 0.05, ***p < 0.001

**Fig. 2:**
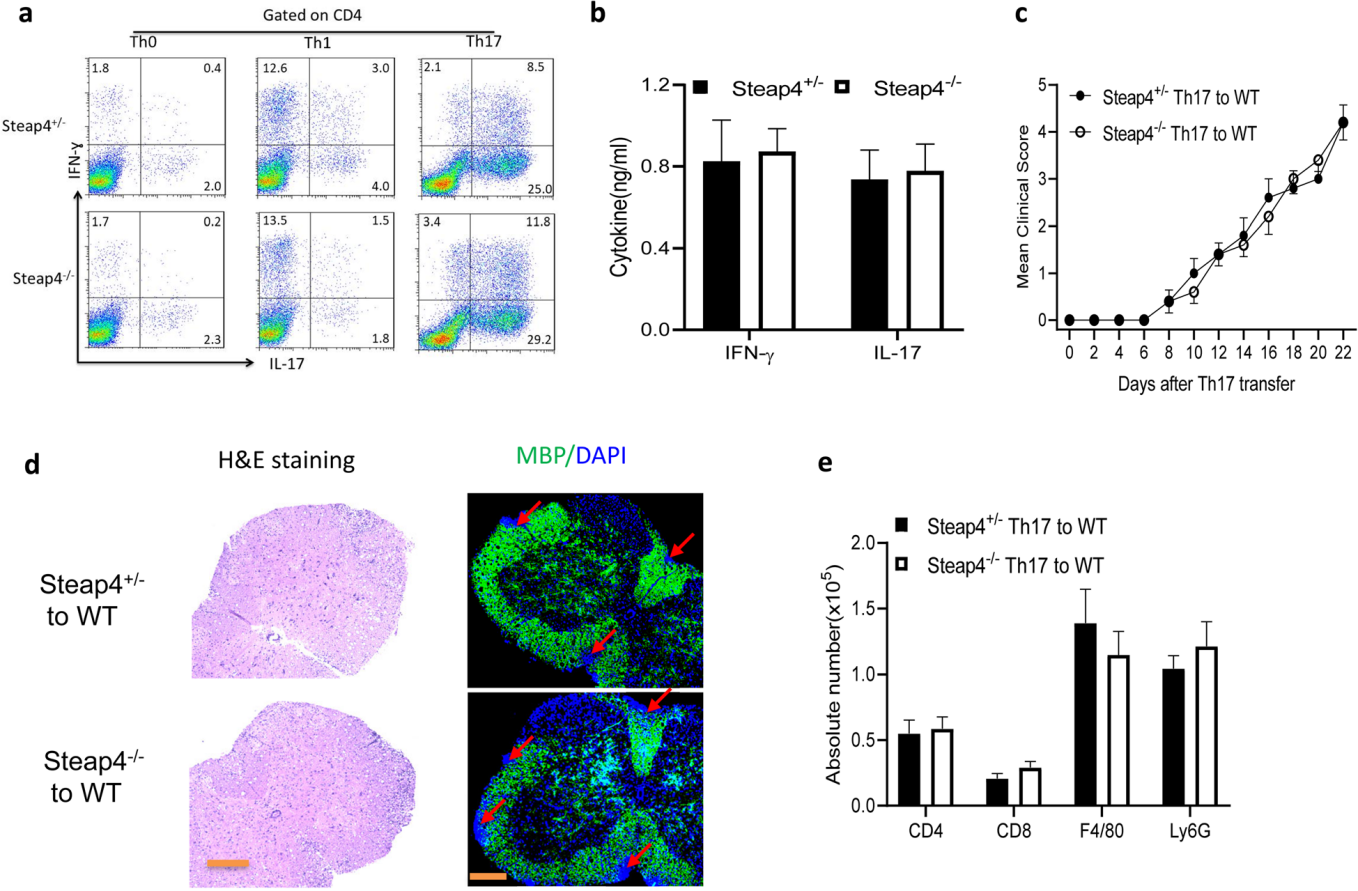
STEAP4^−/−^ T cells are fully encephalitogenic. Mice were immunized by MOG_35–55_ and sacrificed 10 days after immunization. Lymph node cells were harvested and re-stimulated with MOG_35–55_ in DMEM medium alone (Th0), 20 ng/ml IL-12 (Th1), or 20 ng/ml IL-23 (Th17) for 5 days. **a** Data show the frequency of Th1 or Th17 cells under each polarization by intracellular staining. **b** Draining lymph node cells from wild-type mice and STEAP4-deficient mice 10 days after immunization were re-stimulated with MOG_35–55_ in vitro for 5 days, followed by ELISA of IL-17, IFN-γ. **c** MOG_35–55_-specific Th17 cells from wild-type mice and STEAP4-deficient mice were used as donor cells and transferred to naïve wild-type recipient mice. Graph represents the mean clinical score after Th17-cell transfer. **d** H&E and myelin basic protein (MBP) staining of lumber spinal cords at the end point of EAE. **e** Absolute cell numbers of infiltrated immune cells in the brains of recipients of STEAP4^+/-^ Th17 or recipients of STEAP4^−/−^ Th17 cells. Data are representative of three independent experiments. *n* = 5/group in each experiment. Error bars, SEM; *p < 0.05, **p < 0.01

To identify the IL-17 target genes that function as effector molecules in the pathogenesis of EAE, we previously performed a transcriptomic profiling study on spinal cord tissues from wild-type and Act1-deficient (Act1 KO) mice that had received encephalitogenic Th17 cells [31]. Since Act1 deficiency abrogates IL-17 signaling, we reasoned that genes that were induced by encephalitogenic Th17 cells in wild-type but not Act1 knockout spinal cords were likely IL-17 target effector molecules. This led to the identification of *STEAP4* as a top candidate IL-17 target effector during neuroinflammation (Fig. 1a). To determine the role of *STEAP4* in the pathogenesis of EAE, we subjected *STEAP4 knockout* (*STEAP4 KO*) and wild-type littermate control mice to immunization with MOG_35-55_ peptides to induce active *E*AE. Compared to wild-type littermate control, *STEAP4 knockout* mice exhibited markedly delayed disease onset (Fig. 1b). Moreover, while both *STEAP4 knockout* and littermate control mice reached 100% disease incidence 20 days after immunization (Supple. Fig. 1a), the clinical score and mean cumulative EAE scores of the *STEAP4 knockout* mice were lower compared to the controls (Fig. 1b and Supple. Fig. 2a). Consistent with the attenuated disease severity, histopathological analysis showed reduced infiltrating immune cell accumulation and resultant demyelination in spinal cord of the *STEAP4 knockout* mice compared to controls (Fig. 1c). Inflammatory mononuclear cell infiltration in the brain, including T cells, B cells, neutrophils, and macrophages, was consistently reduced in the *STEAP4 knockout* mice (Fig. 1d). Importantly, both the histological analysis and flow cytometric profiling were performed on the EAE mice 20 days post immunization, a time point when all mice had developed disease (Supple. Fig. 1a), indicating that the observed difference was not due to a difference in disease incidence but rather reflected an ameliorated disease severity in *STEAP4 knockout* mice. Collectively, these data suggested that *STEAP4* promotes the pathogenesis of autoimmune encephalomyelitis and exacerbates neuroinflammation.

### *STEAP4* deficiency in T cells does not impact their encephalitogenicity

Current model suggests two distinct stages for the pathogenesis of active immunization-induced EAE. During the initiation stage, immunization induces encephalitogenic Th17 lineage lymphocytes, which subsequently infiltrates the central nervous system to mediate tissue damage in the effector stage of the disease [32, 33]. Given the phenotype of *STEAP4 knockout* mice in the active EAE model (Fig. 1b–d), we first asked whether *STEAP4* plays a role in the differentiation of encephalitogenic Th17 cells. To answer this question, we immunized *STEAP4 knockout* and wild-type littermate control mice with MOG_35-55_ peptides. Ten days after immunization, draining lymph node cells were harvested and culture under a non-polarizing condition (Th0) or condition that promotes the generation of Th1 or Th17 cells (Fig. 2a, b). Flow cytometric and ELISA analyses of the resultant cells indicated that STEAP4 deficiency did not impact the generation of Th1 or Th17 cells (Fig. 2a, b). Consistent with these findings, MOG-reactive Th17 cells from wild-type and *STEAP4 knockout* mice were equally encephalitogenic (Fig. 2c and Supple. Fig. 1b). Mice that received MOG-reactive Th17 cells from *STEAP4 knockout* mice showed comparable levels of demyelination and immune cell infiltration to mice that received wild-type MOG-reactive T cells (Fig. 2d, e). Taken together, the data indicate that *STEAP4* deficiency in T cells does not impact their encephalitogenicity.

### *STEAP4* promotes Th17 cell-induced but not Th1 cell-induced autoimmune encephalomyelitis

**Fig. 3:**
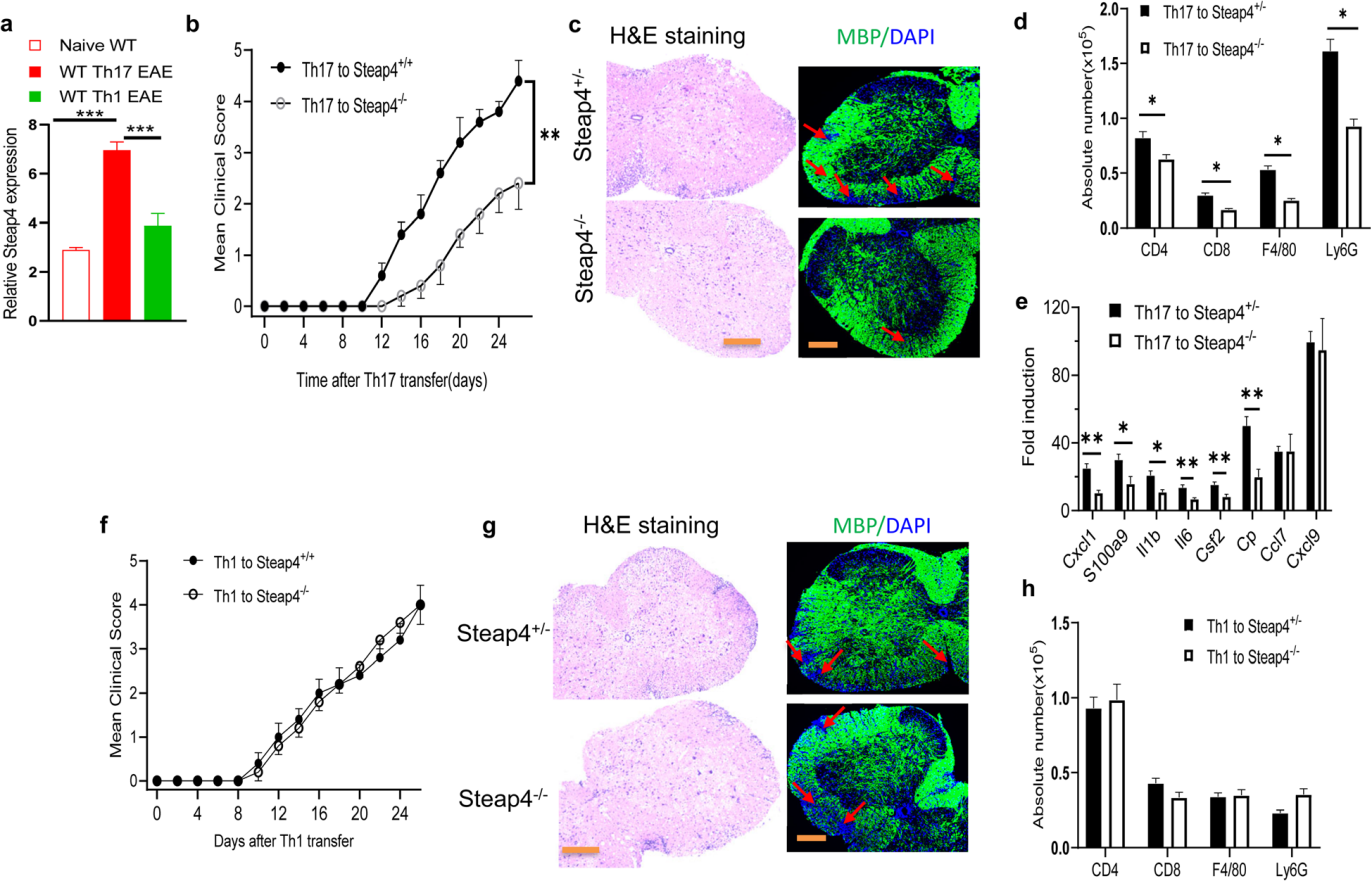
Ablation of STEAP4 ameliorates Th17 cell-induced but not Th1 cell-induced EAE. **a** Microarray analysis of STEAP4 expression in spinal cords from wild-type B6 mice that received MOG_35–55_-reactive Th1 cells or Th17 cells and at the peak of disease, relative to the naïve control mice. Data are re-plotted from GSE97035. **b** MOG_35–55_-specific Th17 cells from wild-type mice were used as donor cells and transferred to naïve STEAP4-deficient mice and the littermate wild-type controls. Graph represents the mean clinical score after Th17-cell transfer. **c** H&E and myelin basic protein (MBP) staining of lumber spinal cords at the end point of EAE. **d** Absolute cell numbers of infiltrated immune cells in the brains of STEAP4^+/−^ and STEAP4^+/−^ recipient mice at the end point of EAE. **e** Real-time PCR analysis of inflammatory gene expression in spinal cords of STEAP4^+/−^ and STEAP4^+/−^ mice transferred with MOG_35–55_-specific Th17 cells. **f** MOG_35–55_-specific Th1 cells from wild-type mice were used as donor cells and transferred to naïve STEAP4-deficient mice and the littermate wild-type controls. Graph represents the mean clinical score after Th1-cell transfer. **g** H&E and MBP staining of the spinal cords from wild-type and STEAP4-deficient mice transferred with Th1 cells at the end point of Th1 EAE. **h** Immune cell infiltration in the brains of wild-type and STEAP4-deficient mice at the end points of Th1 EAE analyzed by flow cytometry. Data are representative of three independent experiments. *n* = 5/group in each experiment. Error bars, SEM; *p < 0.05, **p < 0.01, ***p < 0.001

The expression of *STEAP4* in the EAE spinal cord followed the pattern of clinical scores. *STEAP4* mRNA was induced upon disease onset and it reached maximum at the peak of the disease (Supple. Fig. 3a). Intriguingly, the level of STEAP4 expression trended down among mice that started to recover (Supple. Fig. 3a). We sought to determine whether *STEAP4* participates in the effector stage during the pathogenesis of EAE. Notably, while Th17 cells and its signature cytokine IL-17 is required for EAE induced by active immunization, MOG-reactive, differentiated Th1, and Th17 are both capable of driving neuroinflammation when they are adoptively transferred into sublethally irradiated recipient mice. Importantly, encephalitogenic Th17 but not Th1 cells induced the expression of *STEAP4* in the spinal cord of recipient mice [34, 35] (Fig. 3a, Supple. Fig. 1d), suggesting that *STEAP4* is a Th17-specific effector molecule in the pathogenesis of EAE. To test this idea, we transferred either MOG-reactive Th17 or MOG-reactive Th1 cells into wild-type and *STEAP4 knockout* mice (Fig. 3b–h). Consistent with the specific induction of *STEAP4* by Th17 cells, the disease severity of *STEAP4 knockout* mice was substantially reduced compared to that of the wild-type littermate control (Fig. 3b). The amelioration of EAE symptoms in the *STEAP4 knockout* mice was characterized by delayed onset and peak of disease as well as a dampened cumulative clinical score (Fig. 3b, Supple. Figs. 1 c, 2 b). In line with the clinical score, *STEAP4 knockout* mice also sustained less demyelination in their spinal cords (Fig. 3c) and had less immune cell infiltration in the brain compared to littermate controls (Fig. 3d). The expression of inflammatory cytokine and chemokine expression in the spinal cord was also significantly decreased (Fig. 3e). In contrast, MOG-reactive Th1 cells induced comparable disease in both *STEAP4 knockout* and wild-type littermate controls (Fig. 3f–h, Supple. Fig. 1d). Taken together, the data suggest that *STEAP4* is an effector molecule in Th17- but not Th1-driven EAE.

### *STEAP4* expression in neuroectoderm-derived CNS resident cells is required for the pathogenesis of active EAE

**Fig. 4:**
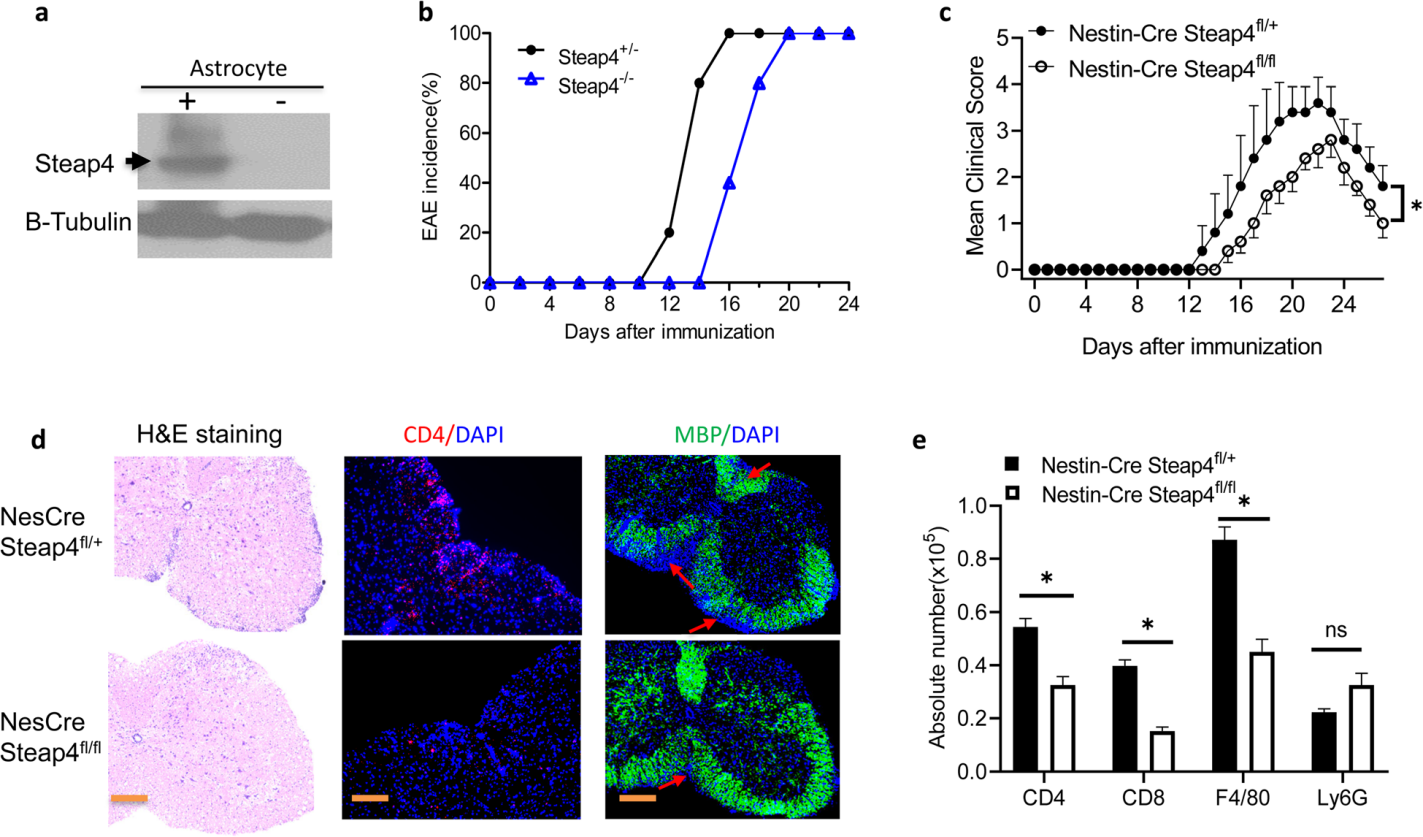
STEAP4 in neuroectoderm-derived cells is critical for actively-induced EAE. **a** Immunoblot analysis for STEAP4 expression in astrocytes from Nestin-Cre STEAP4^fl/+^ and Nestin-Cre STEAP4^fl/fl^ mice. Astrocytes were cultured from brains of neonatal pups. **b** EAE incidence in Nestin-Cre STEAP4^fl/+^ and Nestin-Cre STEAP4^fl/fl^ mice induced by active immunization with MOG35-55. **c** Mean clinical score of EAE in Nestin-Cre STEAP4^fl/+^ and Nestin-Cre STEAP4^fl/fl^ mice induced by active immunization with MOG_35–55_ peptides. **d** H&E, anti-CD4, and MBP staining of spinal cords from Nestin-Cre STEAP4^fl/+^ and Nestin-Cre STEAP4^fl/fl^ mice 22 days after EAE induction. **e** Absolute cell numbers of infiltrated immune cells in the brains of Nestin-Cre STEAP4^fl/+^ and Nestin-Cre STEAP4^fl/fl^ mice 22 days after immunization. Data are representative of three independent experiments. *n* = 5/group in each experiment. Error bars, SEM; *p < 0.05

We have previously shown that IL-17-induced Act1-mediated signaling in neuroectoderm-derived cells in the central nervous system (CNS) is critical for the pathogenesis of EAE [16, 17]. In addition, we reported that IL-17 readily induced STEAP4 expression in a number of tissue cells. These evidences led us to hypothesize that STEAP4 expression in the CNS resident cells is required for active EAE pathogenesis. To test this hypothesis, we generated Nestin-Cre, STEAP4^fl/fl^ mice. Nestin-Cre is expressed in all neuroectoderm-derived cells of the central nervous system, resulting in ablation of LoxP-flanked sequences in all CNS resident cells including astrocytes, neuron and oligodendrocyte lineage cells. Specific STEAP4 ablation was confirmed by western blot in astrocytes from Nestin-Cre STEAP4^fl/+^ and Nestin-Cre STEAP4^fl/fl^ neonatal pups (Fig. 4a). We induced EAE in *Nestin-Cre STEAP4*^*fl/+*^ and *Nestin-Cre STEAP4*^*fl/fl*^ mice by active immunization with MOG_35-55_ peptides. Similar to the phenotype observed in the *STEAP4 global knockout* mice, *Nestin-Cre STEAP4*^*fl/fl*^ mice showed a delayed onset of disease and reduced cumulative clinical scores compared to *Nestin-Cre STEAP4*^*fl/+*^ control mice, but mice in both groups had 100% disease incidence (Fig. 4b, c, Supple. Fig. 2c). The reduction in disease severity was associated with a markedly reduced demyelination and immune cell infiltration (Fig. 4d, e). To determine the contribution of *STEAP4* expression from CNS-resident cells during EAE, we analyzed the level of *STEAP4* expression in spinal cords from *Nestin-Cre STEAP4*^*fl/fl*^ and littermate control EAE mice early at disease onset when mice with comparable disease severity (clinical score = 1~2) were available from both groups. The data showed that Nestin-Cre mediated deletion reduced the expression of *STEAP4* by about 70% in the spinal cord (Supple. Fig. 3B). Furthermore, the expression of *STEAP4* in sorted neutrophil (CD11b Ly6G+) and monocytes (CD11b Ly6C+ Ly6G−) from *Nestin-Cre STEAP4*^*fl/fl*^ and littermate control EAE mice were comparable (Supple. Fig. 3C), indicating that the Cre-expression did not leak into the myeloid-lineage. Together these data suggest that *STEAP4* expression in CNS resident cells is required for the pathogenesis of active EAE.

### *STEAP4* in CNS resident cells promote Th17- but not Th1-induced EAE

**Fig. 5:**
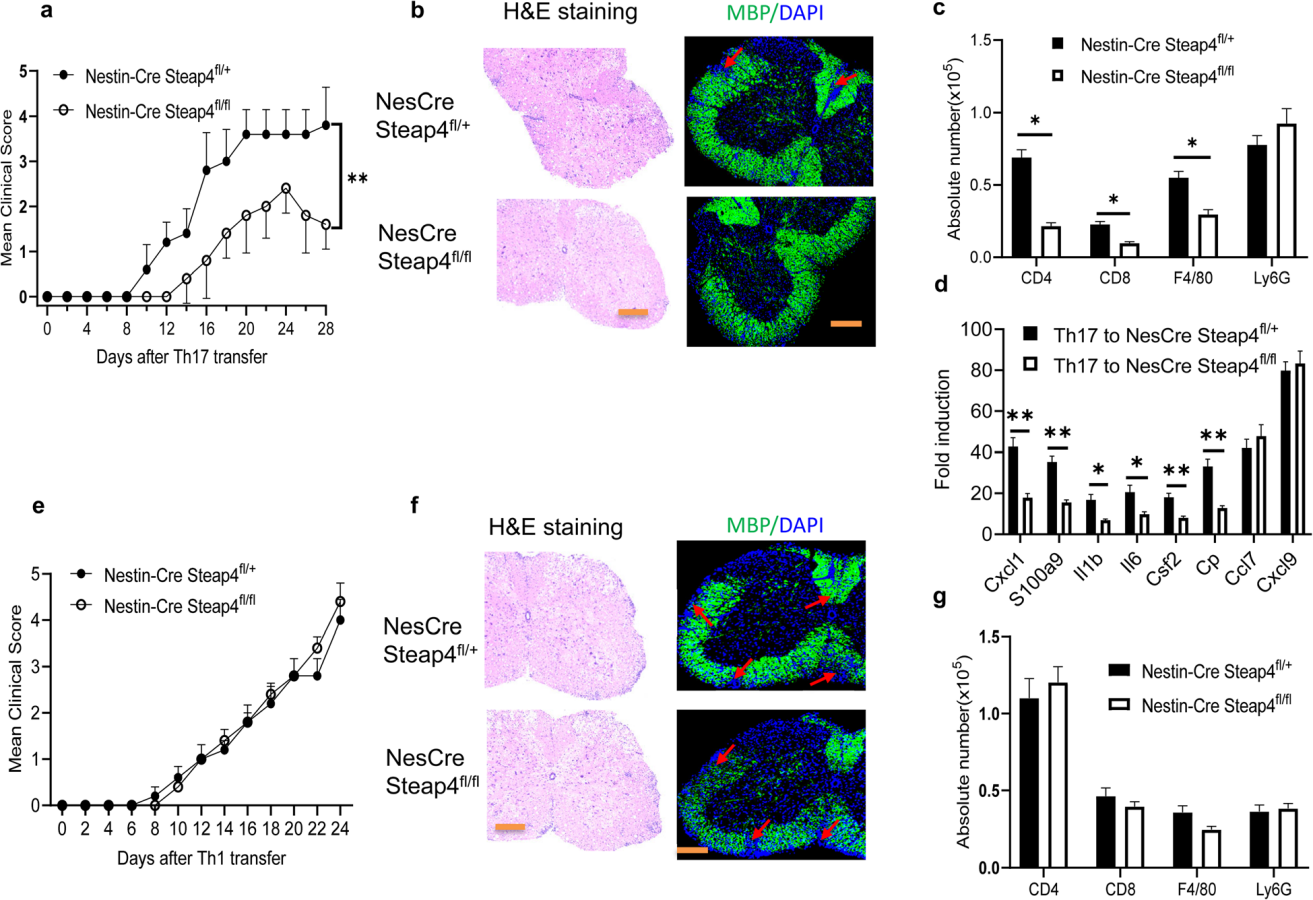
Ablation of STEAP4 in neuroectoderm-derived cells ameliorates Th17 cell-induced EAE. **a** Mean clinical score of EAE in Nestin-Cre STEAP4^fl/+^ and Nestin-Cre Steap4^fl/fl^ mice after MOG_35–55_-specific Th17 cell adoptive transfer. **b** H&E and myelin basic protein (MBP) staining of lumber spinal cords at the end point of EAE. **c** Absolute cell numbers of infiltrated immune cells in the brains of STEAP4^+/−^ and STEAP4^+/−^ recipient mice at the end point of EAE. **d** Real-time PCR analysis of inflammatory gene expression in spinal cords of Steap4^+/−^ and STEAP4^+/−^ mice transferred with MOG_35–55_-specific Th17 cells. **e** MOG_35–55_-specific Th1 cells from wild-type mice were used as donor cells and transferred to naïve STEAP4-deficient mice and the littermate wild-type controls. Graph represents the mean clinical score of EAE after Th1-cell transfer. **f** H&E and MBP staining of the spinal cords from Nestin-Cre STEAP4^fl/+^ and Nestin-Cre STEAP4^fl/fl^ mice at the end point of EAE. **g** Immune cell infiltration in the brains of Nestin-Cre STEAP4^fl/+^ and Nestin-Cre STEAP4^fl/fl^ mice at the end point of EAE analyzed by flow cytometry. Data are representative of three independent experiments. *n* = 5/group in each experiment. Error bars, SEM; *p < 0.05, **p < 0.01

Since our data suggested that *STEAP4* is a specific effector molecule for Th17- but not Th1-induced EAE (Fig. 3), we examined whether the pathogenic impact was predominantly contributed by *STEAP4* expression in CNS resident T cells. Using a similar experimental design, we transferred MOG-reactive Th17 or Th1 cells into Nestin-Cre STEAP4^fl/fl^ mice and littermates Nestin-Cre STEAP4^fl/+^ mice. Consistent with the phenotype observed in the global *STEAP4* knockout mice, the disease onset was much delayed and plateau clinical score was significantly reduced in *Nestin-Cre STEAP4*^*fl/fl*^ mice that received MOG-reactive Th17 cells compared to *Nestin-Cre STEAP4*^*fl/+*^ control recipients though both groups has 100% EAE incidence, consistently the cumulative EAE score was also dramatically reduced in *Nestin-Cre STEAP4*^*fl/fl*^ mice (Fig. 5a, Supple. Figs. 1e, 2d). Demyelination, immune cell infiltration and expression of inflammatory genes were consistently reduced in the *Nestin-Cre STEAP4*^*fl/fl*^ recipients (Fig. 5b–d). In contrast, the onset of disease was simultaneous and the peak clinical score was comparable in Nestin-Cre STEAP4^fl/fl^ and littermate *Nestin-Cre STEAP4*^*fl/+*^ mice that received MOG-reactive Th1 cells, resulting in similar levels of demyelination and immune cell infiltration (Fig. 5e–g, Supple. Fig. 1f). Collectively, the data indicate that *STEAP4* is an effector molecule in CNS resident cells during Th17- but not Th1-induced EAE.

## Discussion

In this study, we identified STEAP4 as a Th17-specific effector molecule that participates and contributes to the pathogenesis of neuroinflammation. While MOG-reactive Th17 cells induced *STEAP4* expression in the spinal cord of EAE mice, STEAP4 deficiency attenuated the onset of EAE induced by active immunization. Our data indicated that the reduced pathogenesis in *STEAP4* knockout mice was unlikely due to the impact of *STEAP4* on T cells, as the induction of MOG-reactive Th17 cells in *STEAP4* knockout mice was intact and those cells exhibited normal encephalitogenicity when transferred into recipient mice, pointing to a role for *STEAP4* in the effector stage of the pathogenesis. Indeed, *STEAP4* knockout mice were relatively resistant to passively induced EAE by MOG-reactive Th17 cells. Importantly, while *STEAP4* was specifically induced by MOG-reactive Th17 cells but not Th1 cells, *STEAP4* deficiency only ameliorated the EAE induced by MOG-reactive Th17 cells but not Th1 cells. This observation is consistent with our previous report showing that *STEAP4* is an IL-17 target gene. This notion was further substantiated by our finding that *STEAP4* expression in the CNS resident cells was critical for both active immunization-induced and MOG-reactive Th17 cells-mediated neuroinflammation.

The identification of a Th17-induced and Th17-specific effector molecule for EAE pathogenesis has major implications. The finding suggest that while both myelin-reactive Th1 and Th17 cells can mediate tissue destruction and demyelination, the underlying molecular process may be drastically different. Indeed, previous study has shown that the disease kinetics and pathologies of EAE elicited by different T helper cell subset or lineages, including Th1, Th17, and Th9 cells [36], have distinct characteristics. Appreciation of the diverging underlying pathogenic mechanism may be critical for our understanding of the broad and extensive heterogeneity in terms of clinical features, genetics, pathogenesis, and responsiveness to treatments in MS patients. In this regard, immunohistochemical analysis of STEAP4 and IL-17 expression in post-mortem brain specimen from MS patients is warranted. In addition, a large number of MS cases are characterized by a relapse-remitting disease course. A key challenge in management of MS is disease relapse. As our data suggest that STEAP4 expression appeared to trend down in mice that started to recover, an important question is whether STEAP4 plays a role in the remission and relapse of neuroinflammation. Future studies using PLP-immunization in SJL mice are required to fully address this question.

A key question raised by our in vivo analysis is how STEAP4 participates and contributes to Th17-mediated demyelination. STEAP4 is an inflammatory metalloreductase that catalyzes the reduction of copper and iron and the oxidation of NADPH. We and others have shown that the expression of STEAP4 promotes the cellular uptake of copper and iron, as only the reduced form of both metal ion are transported across the cellular membrane [24, 25, 37]. We recently reported that increased level of intracellular copper enhances and sustains the activation of NFκB, resulting in elevated production of inflammatory cytokine and chemokines [25]. This STEAP4-mediated induction of chemokine and cytokines would contribute to increased recruitment and activation of immune cells, which eventually lead to tissue damage in the CNS.

On the other hand, the activity of STEAP4 also depletes NADPH and produces NADP+ [24]. NADPH, a potent antioxidant, plays a fundamental role in regulating the intracellular redox balance. NADPH is the ultimate electron donor for most of the detoxifying enzymes that remove harmful reactive oxygen species (ROS), the culprit metabolites for oxidative stress. While moderate level of ROS has been associated with proliferative signals and potentiate inflammatory cytokine production, excessive oxidative stress can lead to cell death. The potential role of STEAP4-mediated ROS production has two implications based on our findings. Microglia, like other immune cells such as macrophages, are likely more equipped to handle oxidative stress. Hence, increased ROS production in microglia may help to amplify the inflammatory response by promoting the production of cytokines and chemokines. However, oligodendrocytes were said to be particularly vulnerable to oxidative stress [38]. Oxidative stress has also been suggested to inhibit oligodendrocyte differentiation [39]. Thus, it is plausible that STEAP4 expression in oligodendrocyte lineage cells may promote direct demyelination and inhibits re-myelination.

In the current study, we primarily focused on the CNS-resident cells because our previous study showed that the CNS-resident cells, and specifically oligodendrocyte progenitor cells, were the target cells for IL-17. While STEAP4 was indeed induced by IL-17 in the CNS-resident cells, myeloid cells, in particular neutrophils, were known to highly express STEAP4 [25, 40]. Notably, the infiltration of macrophages and neutrophils were reduced in the STEAP4 knockout mice in the active EAE model. However, such reduction was less pronounced in the Nestin-Cre STEAP4^fl/fl^ mice, suggesting additional role for STEAP4 in myeloid cells. Nevertheless, whether and how STEAP4 expression in myeloid cells contributes to the pathogenesis of EAE requires further study using cell type specific knockouts.

## Conclusion

In this study, we identified STEAP4 as a Th17-specific effector molecule that participates and contributes to the pathogenesis of neuroinflammation. *STEAP4* knockout mice displayed delayed onset and reduced severity of EAE induced by active immunization. The reduced disease phenotype was not due to any impact of STEAP4 deficiency on myelin reactive T cells. Importantly, *STEAP4* was specifically induced by MOG-reactive Th17 cells but not Th1 cells, *STEAP4* deficiency only ameliorated the EAE induced by MOG-reactive Th17 cells but not Th1 cells. By using Nestin-Cre STEAP4^fl/fl^ mice, we showed that ablation of STEAP4 expression in the resident cells in the central nervous system attenuated disease severity in both active immunization and passive Th17 transfer-induced EAE, thus suggesting a CNS-intrinsic role of STEAP4 in the pathogenesis of autoimmune neuroinflammation. Since STEAP4 is also highly expressed in myeloid cells, whether and how STEAP4 expression in myeloid cells contributes to the pathogenesis of EAE requires further study.

## Supplementary Information

**Additional file 1: Supplementary Figure S1.** EAE incidence after active or passive EAE induction. (a) Steap4^+/-^and Steap4^-/-^ mice were induced to develop EAE by active immunization with MOG_35-55_; Disease incidence are shown. Related to Fig.1 b; related to Fig. 1 b; (b). MOG_35-55_-specific Th17 cells from Steap4^+/-^and Steap4^-/-^ mice were used as donor cells and transferred to naïve wild-type recipient mice. Graph represents EAE incidence after MOG_35-55_-specific Th17-cell transfer, related to Fig. 2 c; (c) MOG_35-55_-specific Th17 cells from wild-type mice were used as donor cells and transferred to naïve Steap4^-/-^ mice and the Steap4^+/-^ littermate controls. Graph represents the EAE incidence after MOG_35-55_-specific Th17-cell transfer, related to Fig. 3 b; (d) MOG_35-55_-specific Th1 cells from wild-type mice were used as donor cells and transferred to naïve Steap4^+/-^and Steap4^-/-^ mice. Graph represents the EAE incidence after Th1-cell transfer, related to Fig. 3 f; (e) EAE incidence in Nestin-Cre Steap4^fl/+^ and Nestin-Cre Steap4^fl/fl^ mice are shown after MOG_35-55_-specific Th17 cell adoptive transfer, related to Fig. 5 a; (f) EAE incidence in Nestin-Cre Steap4^fl/+^ and Nestin-Cre Steap4^fl/fl^ mice are shown after MOG_35-55_-specific Th1 cell adoptive transfer, related to Fig. 5 e. Data are representative of three independent experiments. *n*=5/group in each experiment. *p* values were determined by Log-rank test and shown in each panel.

**Additional file 2: Supplementary Figure S2.** Mean cumulative scores of EAE after active or passive induction. (a) Steap4^+/-^and Steap4^-/-^ mice were induced to develop EAE by active immunization with MOG_35-55_; Mean cumulative scores are shown. Related to Fig. 1 b; (b) Steap4^+/-^and Steap4^-/-^ mice were induced to develop EAE by MOG_35-55_-specific Th17 cell adoptive transfer, Mean cumulative scores are shown. Related to Fig. 3 b; (c) Nestin-Cre Steap4^fl/+^ and Nestin-Cre Steap4^fl/fl^ mice were induced to develop EAE by active immunization with MOG_35-55_; Mean cumulative scores are shown. Related to Fig. 4 c; (d) Nestin-Cre Steap4^fl/+^ and Nestin-Cre Steap4^fl/fl^ mice were induced to develop EAE by MOG_35-55_-specific Th17 cell adoptive transfer, Mean cumulative scores are shown. Related to Fig. 5 a. Data are representative of three independent experiments. *n*=5/group in each experiment. Error bars, SEM. p values were determined by Mann-Whitney test and shown in each panel.

**Additional file 3: Supplementary Figure S3.** Expression pattern and deletion specificity of *Steap4.* (a) Spinal cords were harvested from MOG immunized mice at pre-symptomatic stage (clinical score =0), disease onset (clinical score 2~3), peak of the disease (clinical score 4~5) and remission (clinical score 4~3). Harvested spinal cords were analyzed for *Steap4* expression by RT-PCR. The average 2^-ΔCt^ values of *Steap4* in pre-symptomatic spinal cords were set as 1. The fold changes in spinal cords from disease onset, peak of disease and remission were calculated by divide the 2^-ΔCt^ value of individual biological sample (a spinal cord) by the average 2^-ΔCt^ values of *Steap4* in pre-symptomatic spinal cords, which is set as 1. The *P* value for one-way ANOVA analysis is <0.0001 (smaller than the software limit). Statistically significant *P* values for post hoc two-sided unpaired t test between groups are indicated on the figure. Error bars, SEM (b) Spinal cords from EAE mice of indicated genotype with a clinical score of 1~2 were harvested and analyzed for *Steap4* expression by RT-PCR. The average 2^-ΔCt^ values of *Steap4* in Nestin-Cre Stea4 fl/+ spinal cords were set as 1. Fold change were calculated according the formula described for panel (a). Two-sided unpaired t test was employed to compute the *P* value, which was smaller than 0.0001 beyond software limit. Error bars, SEM. (c-d) Splenocytes from mice of indicated genotype were harvested and sorted by FACS to obtain CD11b+Ly6G+ cells and CD11b+Ly6C+Ly6G- cells. Sorted cells were analyzed for *Steap4* expression by RT-PCR, n=3~5/group in each experiment. The average 2^-ΔCt^ values of *Steap4* in Nestin-Cre Stea4 fl/+ spinal cords were set as 1. Fold change were calculated according the formula described for panel. Error bars, SEM. *P* value by one way ANOVA in (c) is 0.0018. *P* value by one way ANOVA in (d) is 0.0010. Statistically significant *P* values for post hoc unpaired two-sided t test between groups are indicated on the figure.

## Data Availability

All datasets and analyses used in the current study are available from the corresponding author on reasonable request.

## Abbreviations

CNS: Central nervous system
EAE: Experimental autoimmune encephalomyelitis
MS: Multiple sclerosis
Th: T helper cell
H&E: Hematoxylin and eosin
MBP: Myelin basic protein
MOG: Myelin oligodendrocyte glycoprotein
Act1: Nuclear factor NF-kappa-B activator 1
STEAP4: Six transmembrane epithelial antigen of prostate
ELISA: Enzyme-linked immunosorbent assay
