# Supplementary material for: STEAP4 expression in CNS resident cells promotes Th17 cell-induced autoimmune encephalomyelitis
Source: J Neuroinflammation. 2021 Apr 20;18:98. doi: 10.1186/s12974-021-02146-7 (PMC8059164; doi:10.1186/s12974-021-02146-7)
Supplement: Supplementary file 1 — Additional file 1: Supplementary Figure S1. EAE incidence after active or passive EAE induction. (a) Steap4+/-and Steap4-/- mice were induced to develop EAE by active immunization with MOG35-55; Disease incidence are shown. Related to Fig.1 b; related to Fig. 1 b; (b). MOG35-55-specific Th17 cells from Steap4+/-and Steap4-/- mice were used as donor cells and transferred to naïve wild-type recipient mice. Graph represents EAE incidence after MOG35-55-specific Th17-cell transfer, related to Fig. 2 c; (c) MOG35-55-specific Th17 cells from wild-type mice were used as donor cells and transferred to naïve Steap4-/- mice and the Steap4+/- littermate controls. Graph represents the EAE incidence after MOG35-55-specific Th17-cell transfer, related to Fig. 3 b; (d) MOG35-55-specific Th1 cells from wild-type mice were used as donor cells and transferred to naïve Steap4+/-and Steap4-/- mice. Graph represents the EAE incidence after Th1-cell transfer, related to Fig. 3 f; (e) EAE incidence in Nestin-Cre Steap4fl/+ and Nestin-Cre Steap4fl/fl mice are shown after MOG35-55-specific Th17 cell adoptive transfer, related to Fig. 5 a; (f) EAE incidence in Nestin-Cre Steap4fl/+ and Nestin-Cre Steap4fl/fl mice are shown after MOG35-55-specific Th1 cell adoptive transfer, related to Fig. 5 e. Data are representative of three independent experiments. n=5/group in each experiment. p values were determined by Log-rank test and shown in each panel. [file 12974_2021_2146_MOESM1_ESM.docx]

**Additional Files:**

**Additional file 1: Supplementary Figure 1**

**
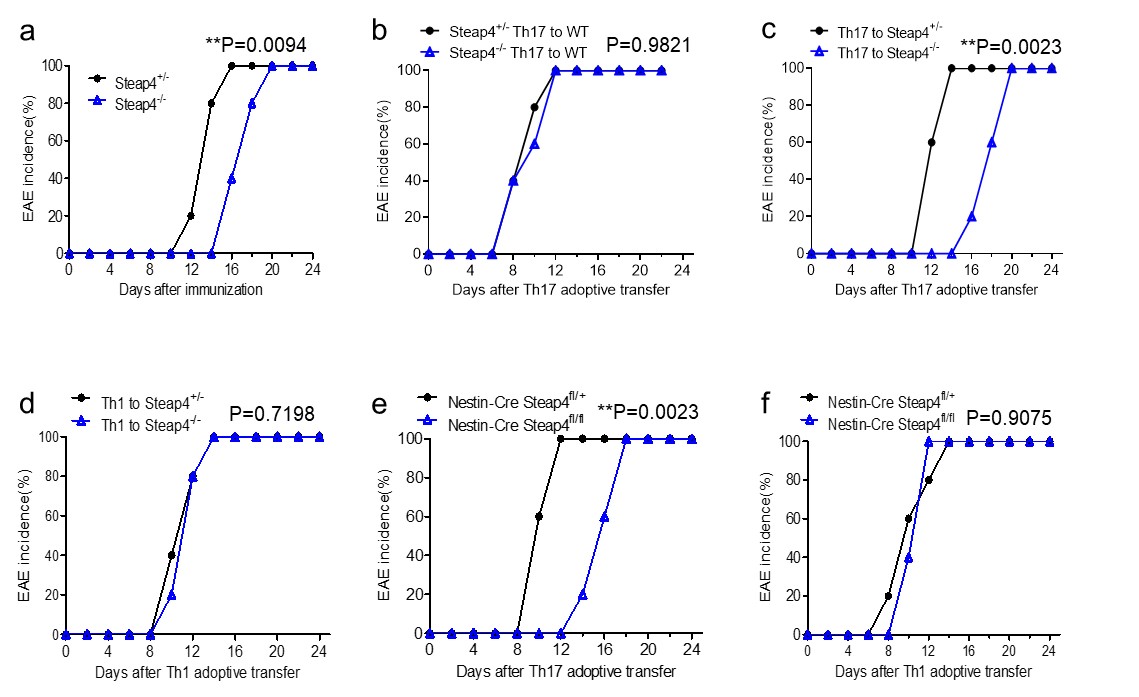
**

**Supplementary Figure 1: EAE incidence after active or passive EAE induction**

(a) Steap4^+/-^and Steap4^-/-^ mice were induced to develop EAE by active immunization with MOG_35-55_; Disease incidence are shown. Related to Fig.1b; related to Fig.1b; (b). MOG_35-55_-specific Th17 cells from Steap4^+/-^and Steap4^-/-^ mice were used as donor cells and transferred to naïve wild-type recipient mice. Graph represents EAE incidence after MOG_35-55_-specific Th17-cell transfer, related to Fig.2c; (c) MOG_35-55_-specific Th17 cells from wild-type mice were used as donor cells and transferred to naïve Steap4^-/-^ mice and the Steap4^+/-^ littermate controls. Graph represents the EAE incidence after MOG_35-55_-specific Th17-cell transfer, related to Fig.3b; (d) MOG_35-55_-specific Th1 cells from wild-type mice were used as donor cells and transferred to naïve Steap4^+/-^and Steap4^-/-^ mice. Graph represents the EAE incidence after Th1-cell transfer, related to Fig. 3f; (e) EAE incidence in Nestin-Cre Steap4^fl/+^ and Nestin-Cre Steap4^fl/fl^ mice are shown after MOG_35-55_-specific Th17 cell adoptive transfer, related to Fig.5a; (f) EAE incidence in Nestin-Cre Steap4^fl/+^ and Nestin-Cre Steap4^fl/fl^ mice are shown after MOG_35-55_-specific Th1 cell adoptive transfer, related to Fig.5e. Data are representative of three independent experiments. n=5/group in each experiment. p values were determined by Log-rank test and shown in each panel.

**Additional file 2: Supplementary Figure 2**

**
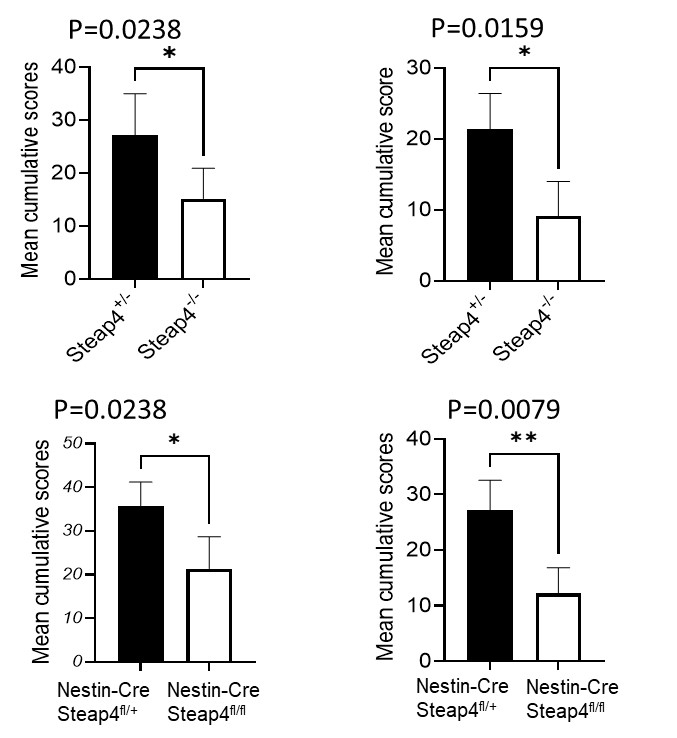
**

**Supplementary Figure 2: Mean cumulative scores of EAE after active or passive induction**

(a) Steap4^+/-^and Steap4^-/-^ mice were induced to develop EAE by active immunization with MOG_35-55_; Mean cumulative scores are shown. Related to Fig.1b; (b) Steap4^+/-^and Steap4^-/-^ mice were induced to develop EAE by MOG_35-55_-specific Th17 cell adoptive transfer, Mean cumulative scores are shown. Related to Fig.3b; (c) Nestin-Cre Steap4^fl/+^ and Nestin-Cre Steap4^fl/fl^ mice were induced to develop EAE by active immunization with MOG_35-55_; Mean cumulative scores are shown. Related to Fig.4c; (d) Nestin-Cre Steap4^fl/+^ and Nestin-Cre Steap4^fl/fl^ mice were induced to develop EAE by MOG_35-55_-specific Th17 cell adoptive transfer, Mean cumulative scores are shown. Related to Fig.5a. Data are representative of three independent experiments. n=5/group in each experiment. Error bars, SEM. p values were determined by Mann-Whitney test and shown in each panel.

**Additional file 3: Supplementary Figure 3**

**
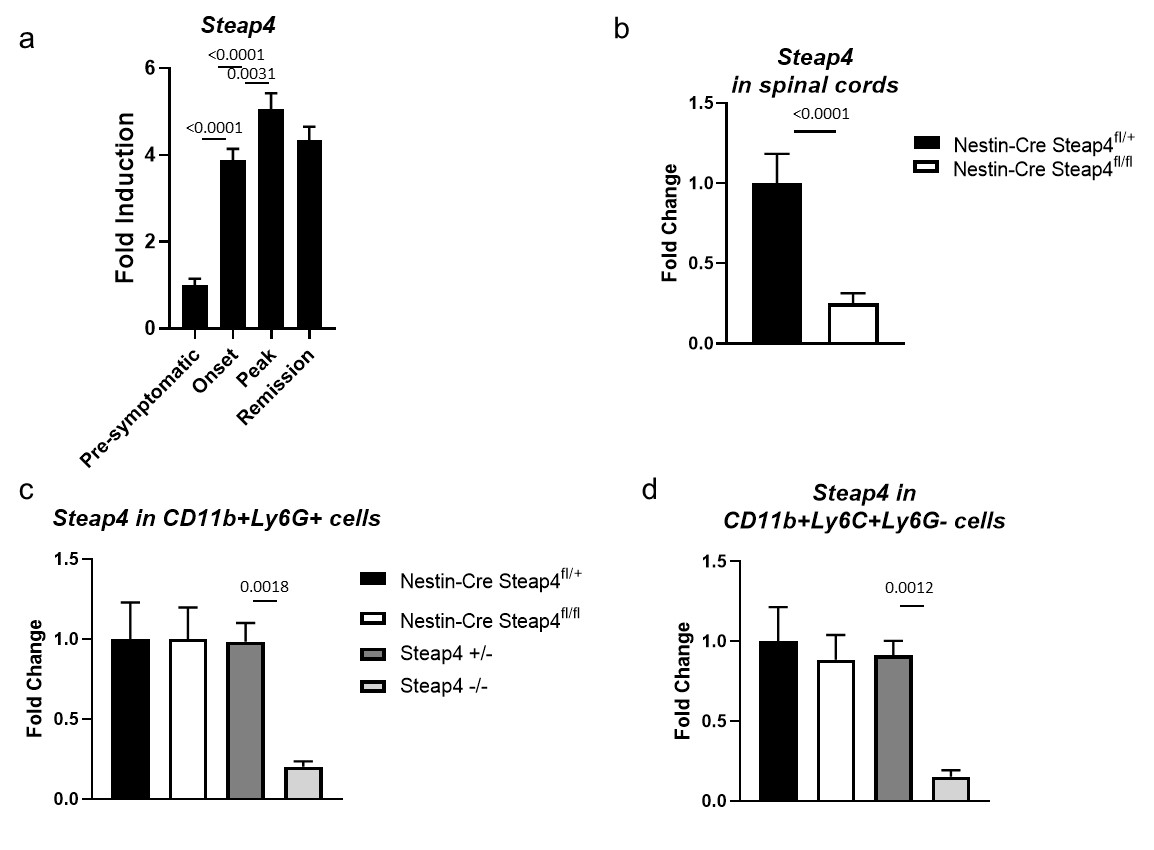
**

**Supplementary Figure 3. Expression pattern and deletion specificity of *Steap4.***

(a) Spinal cords were harvested from MOG immunized mice at pre-symptomatic stage (clinical score =0), disease onset (clinical score 2~3), peak of the disease (clinical score 4~5) and remission (clinical score 4~3). Harvested spinal cords were analyzed for *Steap4* expression by RT-PCR. The average 2^-ΔCt^ values of *Steap4* in pre-symptomatic spinal cords were set as 1. The fold changes in spinal cords from disease onset, peak of disease and remission were calculated by divide the 2^-ΔCt^ value of individual biological sample (a spinal cord) by the average 2^-ΔCt^ values of *Steap4* in pre-symptomatic spinal cords, which is set as 1. The P value for one-way ANOVA analysis is <0.0001 (smaller than the software limit). Statistically significant P values for post hoc two-sided unpaired t test between groups are indicated on the figure. Error bars, SEM (b) Spinal cords from EAE mice of indicated genotype with a clinical score of 1~2 were harvested and analyzed for *Steap4* expression by RT-PCR. The average 2^-ΔCt^ values of *Steap4* in Nestin-Cre Stea4 fl/+ spinal cords were set as 1. Fold change were calculated according the formula described for panel (a). Two-sided unpaired t test was employed to compute the P value, which was smaller than 0.0001 beyond software limit. Error bars, SEM. (c-d) Splenocytes from mice of indicated genotype were harvested and sorted by FACS to obtain CD11b+Ly6G+ cells and CD11b+Ly6C+Ly6G- cells. Sorted cells were analyzed for *Steap4* expression by RT-PCR, n=3~5/group in each experiment. The average 2^-ΔCt^ values of *Steap4* in Nestin-Cre Stea4 fl/+ spinal cords were set as 1. Fold change were calculated according the formula described for panel. Error bars, SEM. P value by one way ANOVA in (c) is 0.0018. P value by one way ANOVA in (d) is 0.0010. Statistically significant P values for post hoc unpaired two-sided t test between groups are indicated on the figure.
