# Supplementary material for: STEAP4 expression in CNS resident cells promotes Th17 cell-induced autoimmune encephalomyelitis
Source: J Neuroinflammation. 2021 Apr 20;18:98. doi: 10.1186/s12974-021-02146-7 (PMC8059164; doi:10.1186/s12974-021-02146-7)
Supplement: Supplementary file 2 — Additional file 2: Supplementary Figure S2. Mean cumulative scores of EAE after active or passive induction. (a) Steap4+/-and Steap4-/- mice were induced to develop EAE by active immunization with MOG35-55; Mean cumulative scores are shown. Related to Fig. 1 b; (b) Steap4+/-and Steap4-/- mice were induced to develop EAE by MOG35-55-specific Th17 cell adoptive transfer, Mean cumulative scores are shown. Related to Fig. 3 b; (c) Nestin-Cre Steap4fl/+ and Nestin-Cre Steap4fl/fl mice were induced to develop EAE by active immunization with MOG35-55; Mean cumulative scores are shown. Related to Fig. 4 c; (d) Nestin-Cre Steap4fl/+ and Nestin-Cre Steap4fl/fl mice were induced to develop EAE by MOG35-55-specific Th17 cell adoptive transfer, Mean cumulative scores are shown. Related to Fig. 5 a. Data are representative of three independent experiments. n=5/group in each experiment. Error bars, SEM. p values were determined by Mann-Whitney test and shown in each panel. [file 12974_2021_2146_MOESM2_ESM.docx]

**Additional file 2: Supplementary Figure 2**

**
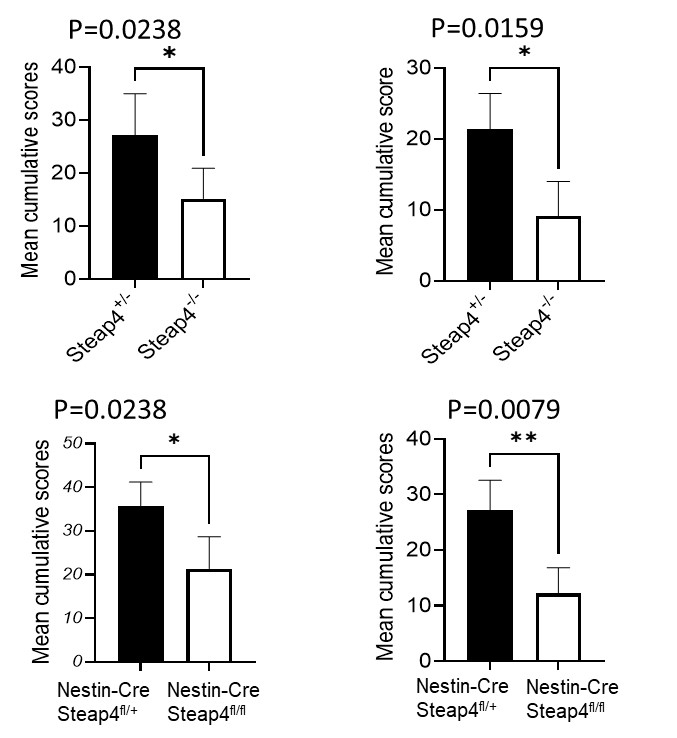
**

**Supplementary Figure 2: Mean cumulative scores of EAE after active or passive induction**

(a) Steap4^+/-^and Steap4^-/-^ mice were induced to develop EAE by active immunization with MOG_35-55_; Mean cumulative scores are shown. Related to Fig.1b; (b) Steap4^+/-^and Steap4^-/-^ mice were induced to develop EAE by MOG_35-55_-specific Th17 cell adoptive transfer, Mean cumulative scores are shown. Related to Fig.3b; (c) Nestin-Cre Steap4^fl/+^ and Nestin-Cre Steap4^fl/fl^ mice were induced to develop EAE by active immunization with MOG_35-55_; Mean cumulative scores are shown. Related to Fig.4c; (d) Nestin-Cre Steap4^fl/+^ and Nestin-Cre Steap4^fl/fl^ mice were induced to develop EAE by MOG_35-55_-specific Th17 cell adoptive transfer, Mean cumulative scores are shown. Related to Fig.5a. Data are representative of three independent experiments. n=5/group in each experiment. Error bars, SEM. p values were determined by Mann-Whitney test and shown in each panel.
