# Supplementary material for: STEAP4 expression in CNS resident cells promotes Th17 cell-induced autoimmune encephalomyelitis
Source: J Neuroinflammation. 2021 Apr 20;18:98. doi: 10.1186/s12974-021-02146-7 (PMC8059164; doi:10.1186/s12974-021-02146-7)
Supplement: Supplementary file 3 — Additional file 3: Supplementary Figure S3. Expression pattern and deletion specificity of Steap4. (a) Spinal cords were harvested from MOG immunized mice at pre-symptomatic stage (clinical score =0), disease onset (clinical score 2~3), peak of the disease (clinical score 4~5) and remission (clinical score 4~3). Harvested spinal cords were analyzed for Steap4 expression by RT-PCR. The average 2-ΔCt values of Steap4 in pre-symptomatic spinal cords were set as 1. The fold changes in spinal cords from disease onset, peak of disease and remission were calculated by divide the 2-ΔCt value of individual biological sample (a spinal cord) by the average 2-ΔCt values of Steap4 in pre-symptomatic spinal cords, which is set as 1. The P value for one-way ANOVA analysis is <0.0001 (smaller than the software limit). Statistically significant P values for post hoc two-sided unpaired t test between groups are indicated on the figure. Error bars, SEM (b) Spinal cords from EAE mice of indicated genotype with a clinical score of 1~2 were harvested and analyzed for Steap4 expression by RT-PCR. The average 2-ΔCt values of Steap4 in Nestin-Cre Stea4 fl/+ spinal cords were set as 1. Fold change were calculated according the formula described for panel (a). Two-sided unpaired t test was employed to compute the P value, which was smaller than 0.0001 beyond software limit. Error bars, SEM. (c-d) Splenocytes from mice of indicated genotype were harvested and sorted by FACS to obtain CD11b+Ly6G+ cells and CD11b+Ly6C+Ly6G- cells. Sorted cells were analyzed for Steap4 expression by RT-PCR, n=3~5/group in each experiment. The average 2-ΔCt values of Steap4 in Nestin-Cre Stea4 fl/+ spinal cords were set as 1. Fold change were calculated according the formula described for panel. Error bars, SEM. P value by one way ANOVA in (c) is 0.0018. P value by one way ANOVA in (d) is 0.0010. Statistically significant P values for post hoc unpaired two-sided t test between groups are indicated on the figure. [file 12974_2021_2146_MOESM3_ESM.docx]

**Additional file 3: Supplementary Figure 3**

**
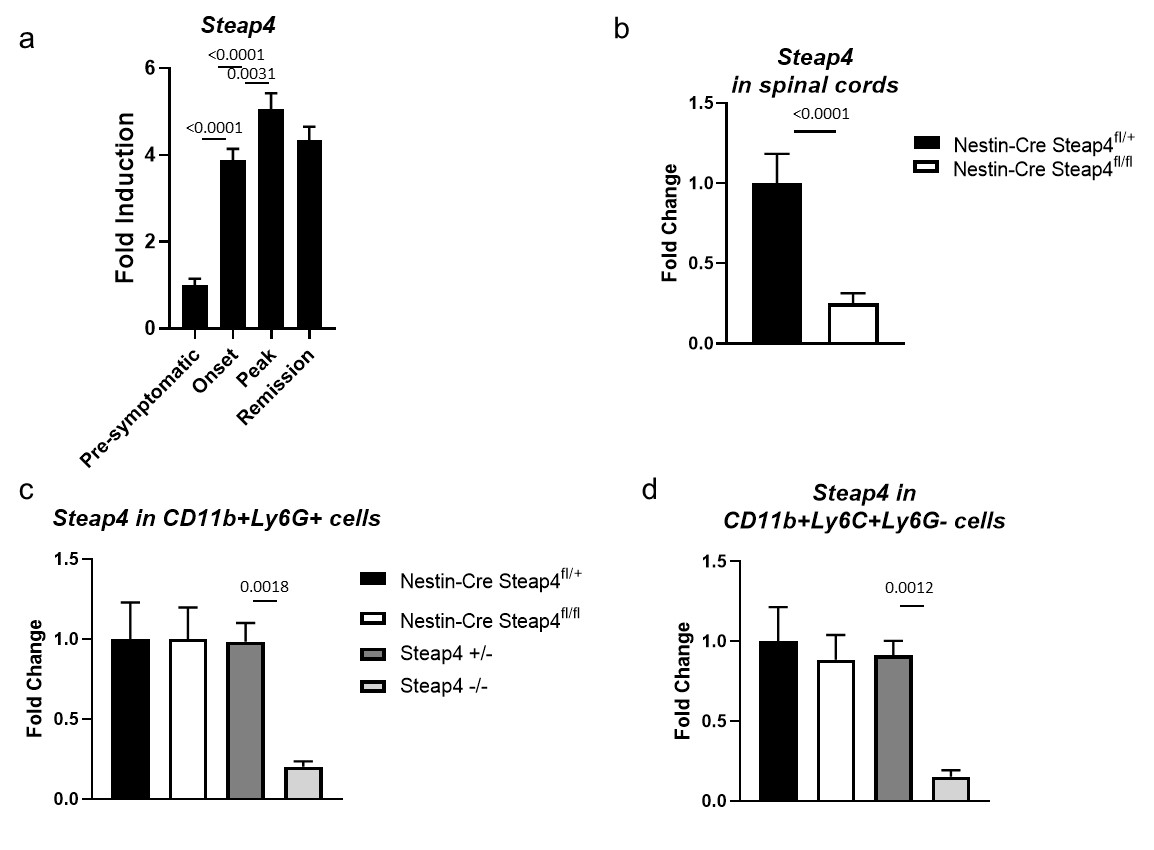
**

**Supplementary Figure 3. Expression pattern and deletion specificity of *Steap4.***

(a) Spinal cords were harvested from MOG immunized mice at pre-symptomatic stage (clinical score =0), disease onset (clinical score 2~3), peak of the disease (clinical score 4~5) and remission (clinical score 4~3). Harvested spinal cords were analyzed for *Steap4* expression by RT-PCR. The average 2^-ΔCt^ values of *Steap4* in pre-symptomatic spinal cords were set as 1. The fold changes in spinal cords from disease onset, peak of disease and remission were calculated by divide the 2^-ΔCt^ value of individual biological sample (a spinal cord) by the average 2^-ΔCt^ values of *Steap4* in pre-symptomatic spinal cords, which is set as 1. The P value for one-way ANOVA analysis is <0.0001 (smaller than the software limit). Statistically significant P values for post hoc two-sided unpaired t test between groups are indicated on the figure. Error bars, SEM (b) Spinal cords from EAE mice of indicated genotype with a clinical score of 1~2 were harvested and analyzed for *Steap4* expression by RT-PCR. The average 2^-ΔCt^ values of *Steap4* in Nestin-Cre Stea4 fl/+ spinal cords were set as 1. Fold change were calculated according the formula described for panel (a). Two-sided unpaired t test was employed to compute the P value, which was smaller than 0.0001 beyond software limit. Error bars, SEM. (c-d) Splenocytes from mice of indicated genotype were harvested and sorted by FACS to obtain CD11b+Ly6G+ cells and CD11b+Ly6C+Ly6G- cells. Sorted cells were analyzed for *Steap4* expression by RT-PCR, n=3~5/group in each experiment. The average 2^-ΔCt^ values of *Steap4* in Nestin-Cre Stea4 fl/+ spinal cords were set as 1. Fold change were calculated according the formula described for panel. Error bars, SEM. P value by one way ANOVA in (c) is 0.0018. P value by one way ANOVA in (d) is 0.0010. Statistically significant P values for post hoc unpaired two-sided t test between groups are indicated on the figure.
